# Efficacy and safety of 3‐n‐butylphthalide combined with endovascular treatment in acute ischemic stroke due to large vessel occlusion

**DOI:** 10.1111/cns.13978

**Published:** 2022-10-02

**Authors:** Shuai Liu, Fengli Li, Jie Yang, Dongjie Xie, Chengsong Yue, Weidong Luo, Jinrong Hu, Jiaxing Song, Linyu Li, Jiacheng Huang, Chenhao Zhao, Zili Gong, Qingwu Yang, Wenjie Zi

**Affiliations:** ^1^ Department of Neurology, Xinqiao Hospital and The Second Affiliated Hospital Army Medical University (Third Military Medical University) Chongqing China; ^2^ Department of Neurology, Chongqing Institute for Brain and Intelligence Guangyang Bay Laboratory Chongqing China

**Keywords:** butylphthalide, endovascular treatment, large vessel occlusion, stroke

## Abstract

**Background:**

The drug 3‐*n*‐butylphthalide (NBP) was developed and approved in China, where it has been used to treat ischemic cerebrovascular diseases. It is also considered to have a neuroprotective effect. This study aimed to evaluate whether NBP combined with endovascular treatment (EVT) can improve the clinical outcome and safety in patients with acute ischemic stroke (AIS) due to large vessel occlusion (LVO).

**Methods:**

Data from three studies of patients treated with EVT for AIS due to LVO were combined in this study. Patients of LVO undergoing EVT were dichotomized into NBP and non‐NBP subgroups. The primary efficacy outcome was the shift of the modified Rankin Scale (mRS) score at 90 days. The secondary efficacy outcome included favorable functional outcomes, functional independence, and excellent outcome (defined as an mRS score of 3 or less) at 90 days. Safety outcomes included mortality within 90 days and symptomatic intracranial hemorrhage (sICH) within 48 h.

**Results:**

A total of 1820 patients undergoing EVT were included in this study; 628 (37.5%) patients received NBP treatment, whereas 1138 (62.5%) did not. After adjusting for multiple factors, NBP was associated with the improvement of functional outcomes at 90 days (adjusted common odds ratio [OR]: 1.503; 95% confidence interval (CI): 1.254–1.801; *p* < 0.001). NBP was associated with a higher rate of 90‐day favorable outcomes (adjusted OR: 1.589; 95% CI: 1.251–2.020; *p* < 0.001) and a lower rate of 90‐day mortality (adjusted OR: 0.486 [95% CI: 0.372–0.635]; *p* < 0.001). sICH occurred in 74 of 682 (10.9%) patients in the NBP group and 155 of 1126 (13.8%) patients in the non‐NBP group; no statistical difference was detected (adjusted OR: 0.787 [95% CI: 0.567–1.092]; *p* = 0.152).

**Conclusion:**

Among patients with AIS due to LVO, NBP combined with EVT is associated with better functional outcomes and reduced mortality risk without increasing the risk of sICH.

## Graphical abstract

Among patients with acute ischemic stroke due to LVO， butylphthalide combined with EVT is associated with better functional outcomes and reduced mortality risk without increasing the risk of symptomatic intracranial hemorrhage.

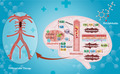

## 1 INTRODUCTION

Endovascular treatment (EVT) has made great progress in recent years as the first‐line treatment for acute intracranial large vessel occlusion (LVO).^1^ Despite successful recanalization rates exceeding 80%, many patients still experience clinical deterioration and tend to have a poor prognosis after successful reperfusion; a phenomenon called futile recanalization (FR).^2^ A meta‐analysis from five famous randomized controlled trials (EXTEND‐IA, MR CLEAN, SWIFT PRIME, ESCAPE, and REVASCAT) found that the incidence of FR after EVT was 54%.^3^ Neuroprotective agents to protect the cerebrum from secondary injury and minimize the incidence of disability have been the focus of stroke research. Unfortunately, almost all previous attempts at developing neuroprotectants for AIS have not shown clinical benefits.^4^, ^5^

Numerous experimental studies have confirmed the effects of butylphthalide (NBP) in protecting mitochondrial function, improving microcirculation dysfunction, attenuating inflammatory responses, and reducing oxidative stress in stroke models.^6^, ^7^, ^8^ Since synthesized NBP (Dl‐3‐*n*‐butylphthalide) was approved for the treatment of AIS in China in 2002, several studies have shown that NBP can reduce cerebral ischemic damage and improve functional outcomes of patients with AIS without EVT.^9^, ^10^, ^11^ However, the efficiency and safety of NBP combined with EVT in patients with LVO remain unclear.

Using a combined nationwide database from three studies, we aimed to evaluate whether NBP combined with EVT could improve functional outcomes in patients with LVO.

## 2 METHODS

### 2.1 Patient selection

Data from three EVT studies of patients with AIS due to LVO have included: the BASILAR study, which is a nationwide registry for acute basilar artery occlusion covering 47 stroke centers in China (http://www.chictr.org.cn，ChiCTR1800014759); the DEVT trial, which is a multicenter open‐label randomized controlled trial with AIS due to anterior LVO from 33 stroke centers in China (http://www.chictr.org.cn, ChiCTR‐IOR‐17013568); and a multicenter study on the safety and efficacy of intravascular therapy for AIS caused by LVO in China (http://www.chictr.org.cn, ChiCTR1800019538). The inclusion criteria were age ≥18 years, LVO of the posterior circulation or anterior circulation confirmed by head digital subtraction angiography, magnetic resonance angiography (MRA), or computer tomography angiography (CTA), and receiving EVTs within 24 h of the estimated time of LVO. The exclusion criteria included premorbid modified Rankin Scale (mRS) of 3 or more, allergy to NBP, cerebral hemorrhage on presentation revealed by CTA or MRA, or missing data on NBP use, and severe liver/renal/heart/lung dysfunction.

### 2.2 NBP treatments

All patients received EVT, including intra‐arterial therapy, medical treatment, balloon angioplasty, stenting, or combination therapy. Early application of NBP in patients after EVT was allowed. NBP was used at the discretion of the physician and started within 24 h of mechanical recanalization. If early NBP was administered, NBP sodium chloride injection (NMPA approval number: H20100041; CSPC Enbipu Pharmaceutical Corporation Ltd.) was administered intravenously (standard: 100 ml, two times per day), slowly dripped over at least 50 min. The treatment was maintained for 14 days, and then NBP capsules (NMPA approval number: H20050299; CSPC Enbipu Pharmaceutical Corporation Ltd.) were orally administrated (standard: 100 mg*2, three times per day) from day 15 to day 90.

### 2.3 Data collection and assessment

Demographic information and baseline clinical characteristics of all eligible patients were extracted from the combined database, including age, sex, history of hypertension, diabetes mellitus, dyslipidemia, coronary artery disease, atrial fibrillation, current smoking status, blood pressure at admission, and National Institutes of Health Stroke Scale (NIHSS) scores at admission. Stroke subtypes were classified according to the Trial of Org 10172 in Acute Stroke (TOAST). The time from stroke onset to groin puncture and time from stroke onset to revascularization was also recorded. The time of stroke onset was the last time the patient had been seen in a normal state. Baseline ischemic injury in the anterior circulation was assessed according to the Alberta Stroke Program Early Computed Tomography (ASPECT) score; injury in the posterior circulation was assessed according to the ASPECT score, as adjusted for the posterior circulation. The quality of reperfusion was evaluated using the modified Thrombolysis in Cerebral Infarction (mTICI) score on the final angiogram. Collateral vessel status was assessed by the American Society of Interventional and Therapeutic Neuroradiology/Society/Society of Interventional Radiology (ASITN/SIR) collateral vessel grading system.

### 2.4 Outcomes

The primary efficiency outcome was the mRS score at 90 days, ranging from 0 (no symptoms) to 6 (death). Follow‐up examinations were performed in outpatient visits or by phone calls by neurologists blinded to the treatment information in the participating centers. Secondary outcomes included a favorable functional outcome (defined as an mRS score of 0–3), functional independence (mRS score of 0–2), and an excellent outcome (mRS score of 0–1). The safety outcomes included symptomatic intracranial hemorrhage (sICH) within 48 h and mortality within 3 months of EVT. The sICH was assessed based on follow‐up CT or MRI according to the Heidelberg Bleeding Classification.

### 2.5 Statistical analysis

For continuous variables, the Kolmogorov–Smirnov test was performed to test the normality first. The continuous variables were described as mean (standard deviation) and median (quartile) according to the normality distribution. We used the Chi‐squared or Fisher exact test for categorical variables and the Student's t‐test or Mann–Whitney U test for continuous variables according to their normality of distribution to compare baseline characteristics and outcomes between the NBP and non‐NBP groups.

Univariable analysis was first performed to identify potential confounding factors. The final entered factors included those with at least marginal significance (*p* < 0.1) between the NBP and the non‐NBP group and those associated with functional outcomes in previous studies.

The shift toward mRS improvement by one score between the NBP and non‐NBP groups was analyzed using multivariable ordinal logistic regression, adjusting for the following variables: age, sex, smoking status, hypertension, hyperlipidemia, systolic blood pressure at admission, diabetes mellitus, baseline NIHSS and ASPECT score, ASITN/SIR grade, time from stroke onset to revascularization, TOAST classification, intravenous thrombolysis (IVT), occlusion sites (anterior circulation/posterior circulation), mTICI score, and the use of antihypertensive drugs. For other dichotomized outcomes, binary logistic regression was performed, adjusting for the same confounding factors. For sensitivity purposes, we used the nearest‐neighbor matching algorithm and set a caliper width of 0.2 to perform 1:1 propensity score matching. The key variables in our database were excluded from the analysis; therefore, missing values were not imputed.

Statistical analysis was performed using SPSS (version 23.0; IBM SPSS Statistics). The figures were drawn using Microsoft Office Excel 2019. We set *p* < 0.05 as the significance level, and all hypothesis tests were two‐sided.

## 3 RESULTS

### 3.1 Baseline characteristics

A total of 1820 patients were included in this study. The median (interquartile range) age was 66 (57–74) years, the baseline NIHSS score was 17 (12–24), and 1150 (63.5%) of the patients were women. The rate of IVT was 25.8%, and 84.1% (1524/1812) of patients achieved successful reperfusion (mTICI 2b‐3). There were 628 (37.5%) and 1138 (62.5%) patients in the NBP and non‐NBP groups, respectively. Compared with the non‐NBP group, patients in the NBP group had a lower baseline NIHSS score (16 [11–22] vs. 18 [13–26] *p* < 0.001), lower systolic blood pressure levels on admission (145 [128–160] vs. 150 [132–166], *p* = 0.001), lower onset to groin puncture time (315 [230–450] vs. 301 [221–413], *p* = 0.020), lower incidence of hypertension (51.5% vs. 62.5%), and a significant difference in ASITN/SIR grade (e.g., 0–1: 42.7% vs. 50.2%, *p* = 0.006). Other baseline characteristics did not differ significantly between the groups (*p* > 0.05) (Table 1).

**TABLE 1:** Baseline characteristics of the study population: patients with acute LVO

Characteristics	All patients			*χ* ^2^/*z* value	*p* value	Propensity score matching				
	No/total No. (%)					No/total No. (%)			*χ* ^2^/*z* value	*p* value
	All	NBP	Non‐NBP			All	NBP	Non‐NBP		
Number of patients	1820	682	1138			1198	599	599		
Age, years, median (IQR)	66 (57–74)	66 (56–73)	66 (58–75)	−1.320	0.187	66 (56.75–74)	66 (57–74)	65 (56–75)	−0.078	0.938
≥66	943/1820 (51.8)	352/682 (51.6)	591/1138 (51.9)	0.018	0.895	610/1198 (50.9)	311/599 (51.9)	299/599 (49.9)	0/0.481	0.488
Male sex, *n* (%)	1150/1820 (63.5)	429/682 (62.9)	726/1138 (63.8)	0.147	0.702	772/1198 (64.4)	377/599 (62.9)	395/599 (65.9)	1.180	0.277
Baseline NIHSS score, median (IQR)	17 (12–24)	16 (11–22)	18 (13–26)	−5.599	0.000	16 (12–23)	16 (11–22)	16 (12–23)	−0.716	0.474
≥17	934/1820 (51.3)	298/682 (43.7)	636/1138 (55.9)	25.375	0.000	567/1198 (47.3)	276/599 (46.1)	291/599 (48.6)	0.763	0.385
ASITN/SIR grade										
0–1	859/1812 (47.4)	290/679 (42.7)	569/1133 (50.2)	10.151	0.006	517/1198 (43.2)	257/599 (42.9)	260/599 (43.4)	0.110	0.947
2	545/1812 (30.1)	217/679 (32.0)	328/1133 (28.9)			374/1198 (31.2)	186/599 (31.1)	188/599 (31.4)		
3–4	408/1812 (22.5)	172/679 (25.3)	236/1133 (20.8)			307/1198 (25.6)	156/599 (26.0)	151/599 (25.2)		
Baseline ASPECT score, median (IQR)^c^	8 (7–10)	8 (7–10)	8 (7–10)	−0.057	0.954	8 (7–10)	8 (7–10)	8 (7–10)	−0.289	0.773
≥8	1102/1781 (61.9)	408/663 (61.5)	694/1118 (62.1)	0.051	0.822	760/1198 (63.4)	378/599 (63.1)	382/599 (63.8)	0.058	0.810
Blood pressure on admission, median (IQR), mmHg	148 (130–164.5)	145 (128–160)	150 (132–166)	−3.248	0.001	145 (130–161)	145 (129.5–161)	145 (130–162)	−0.134	0.893
Medical history, No. (%)										
Hypertension	1044/1820 (57.4)	351/682 (51.5)	693/1138 (62.5)	15.505	0.000	647/1198 (54.0)	329/599 (54.9)	318/599 (53.1)	0.407	0.524
Hyperlipidemia	407/1820 (22.4)	167/682 (24.5)	240/1138 (21.1)	2.835	0.092	278/1198 (23.2)	151/599 (25.2)	127/599 (21.2)	2.698	0.100
Diabetes mellitus	379/1820 (20.8)	133/682 (19.5)	237/1138 (37.5)	1.157	0.282	246/1198 (20.5)	121/599 (20.2)	125/599 (20.9)	0.082	0.775
Smoking	560/1820 (30.8)	211/682 (30.9)	349/1138 (30.7)	0.015	0.904	382/1198 (31.9)	183/599 (30.6)	199/599 (33.2)	0.984	0.321
Ischemic stroke	280/1820 (15.4)	99/682 (14.5)	181/1138 (15.9)	0.631	0.427	183/1198 (15.3)	91/599 (15.2)	92/599 (15.4)	0.006	0.936
Atrial fibrillation	683/1819 (37.5)	248/682 (36.4)	435/1137 (38.3)	0.653	0.419	439/1198 (36.6)	223/599 (37.2)	216/599 (36.1)	0.176	0.675
Treatment profiles										
Onset to groin puncture, median (IQR), min	309 (224–427)	315 (230–450)	301 (221–413)	−2.331	0.020	310 (230–429.5)	320 (227–445.5)	310 (230–430)	−0.237	0.813
Onset to revascularization, median (IQR), min	397 (279–549)	400 (277–565)	393 (280–541)	−0.537	0.591	400 (278–567)	395 (275–545)	406 (285–590)	−1.490	0.136
Groin puncture to revascularization, median (IQR	99 (67–140)	95 (65–140)	100 (69–142)	−1.652	0.099	97.5 (65–140)	95 (65–136)	100 (65–145)	−1.151	0.250
Stroke causative mechanism										
Large artery atherosclerosis	890/1820 (48.9)	333/682 (48.9)	557/1138 (48.9)	1.818	0.403	584/1198 (48.7)	290/599 (48.4)	294/599 (49.1)	0.263	0.877
Cardioembolism	779/1820 (42.8)	285/682 (41.8)	494//1138 (43.4)			507/1198 (42.3)	253/599 (42.2)	254/599 (42.4)		
Other	151/1820 (8.3)	64/682 (8.3)	81//1138 (8.3)			107/1198 (8.9)	56/599 (9.3)	51/599 (8.5)		
General anesthesia, No. (%)	545/1812 (30.1)	198/680 (29.1)	347/1132 (30.7)	0.477	0.526	855/1198 (71.4)	431/599 (72.0)	424/599 (70.8)	0.200	0.655
IV Thrombolysis	470/1820 (25.8)	175/682 (25.7)	295/1138 (25.9)	0.015	0.901	319/1198 (26.6)	163/599 (27.2)	156/599 (26.0)	0.209	0.647
Occlusion sites										
Anterior circulation	1154/1820 (63.4)	438/682 (64.2)	716/1138 (62.9)	0.313	0.576	746/1198 (62.3)	373/599 (62.3)	373/599 (62.3)	0.000	1.000
Posterior circulation	666/1820 (36.6)	244/682 (35.8)	432/1138 (37.1)			452/1198 (37.7)	226/599 (37.7)	226/599 (37.7)		
mTICI score^a^										
0‐2a	288/1812 (15.9)	101/675 (15.0)	187/1137 (16.4)	0.689	0.404	187/1198 (15.6)	87/599 (14.5)	100/599 (16.7)	1.071	0.301
2b or 3	1524/1812 (84.1)	574/675 (85.0)	950/1137 (83.6)			1011/1198 (84.4)	512/599 (85.5)	499/599 (83.3)		
Medications										
Antihypertensive drugs^d^	1023/1813 (56.4)	344/680 (50.6)	679/1133 (59.9)	15.081	0.000	635/1198 (53.0)	323/599 (53.9)	312/599 (52.1)	0.405	0.524
Hypoglycemic drugs^e^	364/1808 (20.1)	129/679 (19.0)	235/1129 (20.8)	0.870	0.351	240/1198 (20.0)	117/599 (19.5)	123/599 (20.5)	0.188	0.665
Lipid‐lowering drugs^f^	393/1814 (21.7)	161/681 (23.6)	232/1133 (20.5)	2.511	0.113	272/1198 (22.7)	147/599 (24.5)	125/599 (20.9)	2.302	0.129
Anticoagulant drugs^g^	661/1806 (36.6)	241/677 (35.6)	420/1129 (37.2)	0.469	0.494	432/1198 (36.1)	221/599 (36.9)	211/599 (35.2)	0.362	0.547

Abbreviations: ASPECTS, Alberta Stroke Program Early CT Score; IQR, interquartile range; IV, intravenous; mTICI, modified thrombolysis in cerebral infarction; NIHSS, National Institutes of Health Stroke Scale.

^a^ Missing data in 8 patients.

^b^ Missing data in 1 patient.

^c^Missing data in 39 patients.

^d^ Missing data in 7 patients.

^e^ Missing data in 12 patients.

^f^ Missing data in 6 patients.

^g^ Missing data in 14 patients.

### 3.2 Primary efficacy outcome

**FIGURE 1:**
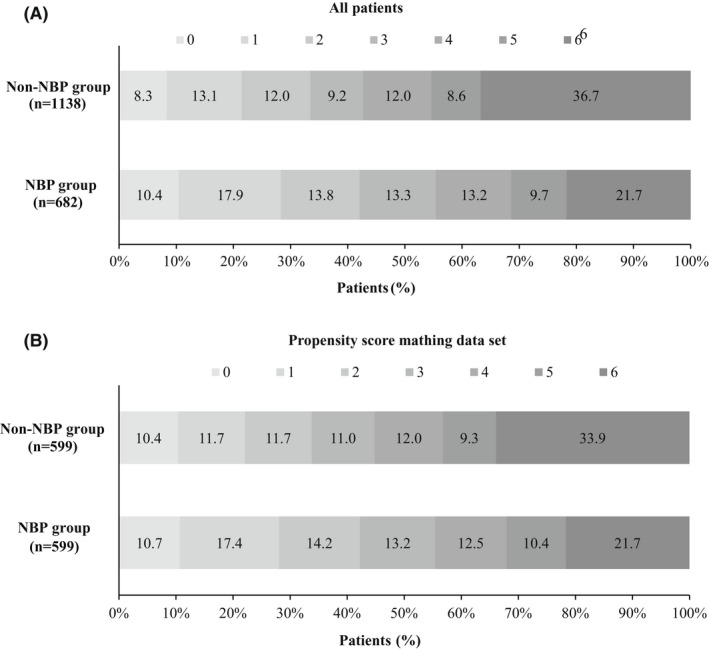
Distribution of the modified Rankin Scale (mRS) at 90 days of the 3‐*n*‐butylphthalide (NBP) group and the non‐NBP group. The distribution of the mRS score of primary outcomes and mortality in both groups among all patients treated with endovascular treatment are presented in (A). The distribution of the mRS score of primary outcomes and mortality in the propensity score matching data set are presented in (B).

The median mRS at 90 days was 3 (2–5) in the NBP group and 4 (2–6) in the non‐NBP group (*p* < 0.001). The common odds ratio (cOR) for improved functional outcomes with NBP was 1.491 (95% confidence interval [CI]: 1.254–1.801) after adjustment for confounding factors (Figure 1 and Table 2).

**TABLE 2:** Efficacy outcomes and safety outcomes

Characteristics	All patients			*χ* ^2^/*z* value	Unadjusted outcome variable value (95% CI)	*p* value	Adjusted value (95% CI)	*p* value	Propensity score matching				
	No/Total No. (%)								No/Total No. (%)			*χ* ^2^/*z* value	*p* value
	All (*n* = 1820)	NBP (*n* = 682)	Non‐NBP (n = 1138)						All (*n* = 1198)	NBP (*n* = 599)	Non‐NBP (*n* = 599)		
Primary efficacy outcomes													
Modified Rankin Scale score at 90d, median (IQR)	4 (2–6)	3 (2–5)	4 (2–6)	−5.976	1.659 (1.403–1.961)	<0.001	1.503 (1.254–1.801)	<0.001	3 (1–6)	3 (1–5)	4 (2–6)	−4.122	<0.001
Secondary efficacy outcomes													
Modified Rankin Scale score at 90d, No. (%)													
0–3, No. (%)	864/1820 (47.5)	378/682 (55.4)	486/1138 (42.7)	27.664	1.668 (1.378–2.020)	<0.001	1.589 (1.251–2.020)	<0.001	600/1198 (50.1)	332/599 (55.4)	268/599 (44.7)	13.675	<0.001
0–2, No. (%)	668/1820 (36.7)	287/682 (42.1)	381/1138 (33.5)	13.583	1.444 (1.187–1.756)	<0.001	1.307 (1.031–1.658)	0.027	455/1198 (38.0)	253/599 (42.2)	202/599 (33.7)	9.217	0.003
0–1, No. (%)	437/1820 (24.0)	193/682 (28.3)	244/1138 (21.4)	10.992	1.446 (1.162–1.799)	0.001	1.362 (1.052–1.763)	0.019	300/1198 (25.0)	168/599 (28.0)	132/599 (22.0)	5.763	0.016
Safety outcome													
Symptomatic intracranial hemorrhage, No. (%)	229/1808 (12.7)	74/682 (10.9)	155/1126 (13.8)	3.263	0.762 (0.568–1.024)	0.071	0.787 (0.567–1.092)	0.152	132/1198 (11.0)	62/599 (10.4)	70/599 (11.7)	0.545	0.460
Mortality at 90 days, No. (%)^a^	566/1820 (31.3)	148/682 (21.7)	418/1138 (36.7)	44.959	0.477 (0.384–0.594)	<0.001	0.486 (0.372–0.635)	<0.001	333/1198 (27.8)	130/599 (21.7)	203/599 (33.9)	22.164	<0.001

Abbreviations: CI, confidence interval; IQR, interquartile range.

^a^ All cause death.

### 3.3 Secondary efficacy outcome

At 90 days, 378 (55.4%) patients in the NBP group compared with 486 (42.7%) patients in the non‐NBP group had a favorable functional outcome (adjusted OR: 1.589, 95% CI: 1.251–2.020, *p* < 0.001; Table 2), 287 (42.1%) patients in the NBP group compared with 381 (33.5%) patients in the non‐NBP group had functional independence (adjusted OR: 1.307, 95% CI: 1.031–1.658, *p* = 0.027; Table 2), and 193 (28.3%) patients in the NBP group compared with 244 (21.4%) patients in the non‐NBP group had excellent functional outcome (adjusted OR: 1.362, 95% CI: 1.052–1.763: *p* = 0.019; Table 2).

### 3.4 Safety outcomes

Overall, sICH occurred in 229 (12.7%) patients: 10.9% in the NBP group and 13.8% in the non‐NBP group (adjusted OR: 0.787, *p* = 0.152), and mortality in the NBP group was 21.7%, which was significantly lower than the 36.7% mortality rate in the non‐NBP group (adjusted OR: 0.486, *p* < 0.001, Table 2).

### 3.5 Propensity score matching analysis

Baseline characteristics achieved balance in the NBP and non‐NBP groups after 1:1 propensity score matching (Table 1). The median mRS at 90 days in the NBP group was 3 [1–5], which was also significantly lower than that of 4 [2–6] in the non‐NBP group (*p* < 0.001). Proportions for 0–1, 0–2, and 0–3 of the mRS at 90 days in the NBP group were still significantly higher than those in the non‐NBP group (respectively, 28.0% vs. 22.0%, *p* = 0.016; 42.2% vs. 33.7%, *p* = 0.003; 55.4% vs. 44.7%, *p* < 0.001).

### 3.6 Subgroup analysis

**FIGURE 2:**
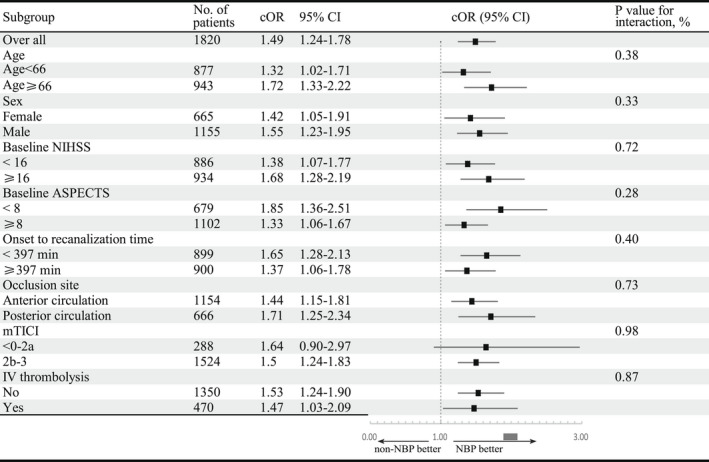
Subgroup analysis of modified Rankin Scale (mRS) at 90 days of the 3‐*n*‐butylphthalide (NBP) group and the non‐NBP group. A forest plot shows the odds of improvement of one point on the modified Rankin scale at 90 days, favoring the NBP group across all prespecified subgroups except mTICI 0‐2a. The thresholds for age, NIHSS, ASPECTS, mTICI, and onset to recanalization time were chosen at the median. ASPECTS, Alberta Stroke Program Early Computed Tomography Score; mTICI, modified Thrombolysis in Cerebral Infarction; NIHSS, National Institute of Health stroke scale

As shown in Figure 2 and Supplementary Figures S2–S9, there was a consistent effect on 90‐day functional outcomes in favor of NBP across any of the prespecified subgroups (defined according to age, sex, baseline NIHSS, baseline ASPECT score, onset to recanalization time, occlusion site, mTICI, and intravenous thrombolysis). The interaction analysis also showed no evidence of heterogeneity in the efficacy of NBP (*p* for interaction >0.05).

## 4 DISCUSSION

This is the largest registry to explore the efficacy and safety of NBP in patients with AIS due to LVO undergoing EVT in the Chinese population. We found that among patients with AIS‐LVO, NBP was significantly associated with a reduction in mortality and improvements in functional outcomes at 90 days.

Butylphthalide can act on multiple pathological pathways in the ischemic tissue and improve the nerve function of patients with AIS. A meta‐analysis of 12 studies involving 1160 patients with ischemic stroke also demonstrated that the combination of NBP and anti‐ischemic stroke drugs used in patients with AIS had a better therapeutic effect than standard drugs alone.^13^ However, whether NBP combined with EVT can improve the clinical prognosis and neurological function of patients with LVO remains unclear.

In this study, patients in the NBP group had higher rates of 90‐day favorable outcomes and functional independence and a lower mortality rate than patients in the non‐NBP group. Furthermore, all prespecified exploratory subgroups based on age, sex, baseline NIHSS, baseline ASPECT score, and intravenous thrombolysis showed results in favor of NBP use, which means that NBP may be a promising neuroprotective agent in the future. Previous studies have shown that NBP is a neuroprotective drug with multiple functions. (1) Reconstructing microcirculation: first, NBP can inhibit arachidonic acid levels and increase prostaglandin I2 (PGI2) and nitric oxide (NO) content in the vascular endothelial cells of the ischemic‐impaired area. PGI2 and NO are factors that can expand cerebrum vessels and improve perfusion in ischemic areas.^8^, ^11^, ^12^ Second, NBP can increase the expression of vascular endothelial growth factor, which increases microvessel density, maintains microvessel integrity, and opens collateral circulation, thus improving brain perfusion in the infarcted area.^9^, ^13^ (2) Protecting mitochondrial function: NBP could enhance Na^+^‐K^+^‐ATPase and Ca^2+^‐ATPase activity, which regulates the transportation of Na^+^ and K^+^ across the nerve cells, maintains cell volume and neuronal excitability, and prevents mitochondrial swelling.^14^, ^15^, ^16^ Moreover, NBP can enhance the activity of cytochrome *c*, which modulates electron transfer in the mitochondrial redox reactions of the respiratory chain, protecting the structure and function of the mitochondria.^17^, ^18^ Thus, NBP improves the cellular tolerance to ischemic stroke, leading to an overall improvement in postischemic neurological impairment. (3) Inhibiting inflammatory responses and antioxidant stress: first, NBP can exert its anti‐inflammatory effect by upregulating the expression of hepatocyte growth factor and downregulating the expression of Toll‐like receptor 4.^19^, ^20^ Second, NBP reduces oxidative stress by increasing superoxide dismutase activity in the cerebral ischemic area, reducing reactive oxygen species and malondialdehyde levels.^21^, ^22^ Third, NBP can reduce the level of phosphorylated ERK, thereby inhibiting the inflammatory response and demonstrating an important neuroprotective effect.^23^

Patients with basilar artery occlusion (BAO) present with particularly severe symptoms on admission compared with those with anterior LVO. From our baseline differences, patients with BAO were younger, more often male, had a higher baseline NIHSS score and a lower ASPECTS score, and so on (Supplementary Table S1), which is consistent with previous studies.^24^ Although patients with BAO had a worse outcome, after adjusting for baseline differences, there was no heterogeneity between BAO and acute anterior LVO regarding the mRS score and mortality at 90 days (Tables S2 and S3 and Figure S1), which further validates our results. On the basis of mechanical recanalization, NBP may have a potential protective effect on neurovascular units, thus improving the prognosis both in patients with BAO or anterior LVO.

Our study was innovative because it targeted a population with AIS due to LVO undergoing EVT, which is relatively common and consistently predicts poor outcomes. Therefore, it is important to timely treat these events. Successful reperfusion via EVTs promotes the likelihood of a good functional outcome, and neuroprotective drugs may be more effective when complete reperfusion is achieved.^25^ Therefore, we chose patients who underwent EVT. Excitotoxicity, oxidative stress, and inflammation have been identified as key factors in ischemic cell death within the penumbra. Given the failures of single‐target neuroprotective drug studies, NBP, a multi‐target drug, is promising for improving clinical outcomes. Multitarget drug action may be key in achieving neuroprotection.

There are still a few important limitations to our study. First, as an observational study, bias in patient selection and treatment procedures was inevitable. Although confounding factors could be adjusted by propensity score matching or multivariate logistic analyses, systematic differences between treatment groups still existed. Second, the works of the literature suggest that the hypoperfusion intensity ratio,^26^ the level of collateral flow,^27^ and net water uptake^28^ and so on are all useful imaging biomarkers that are associated with treatment effects in acute stroke. Unfortunately, such useful information was lacking in our study. Therefore, relevant markers will be added to our study in the future to further testify to the neuroprotective effect of butylphthalide on LVO patients. In addition, the best time window for neuroprotection is 4–6 h after stroke onset, so whether NBP should be administered before EVT may be considered in future studies.

## 5 CONCLUSION

In conclusion, NBP may improve 90‐day functional outcomes and reduce mortality risk in patients with LVO treated with EVT. Further randomized clinical trials are necessary to confirm the NBP results.

### AUTHOR CONTRIBUTORS

QY and WZ take responsibility for the integrity of the data and the accuracy of the data analysis; SL, FL, and JY were involved in the design of the study, the first draft, and contributed equally to this paper. All authors did the acquisition, statistical analysis, or interpretation of the data. The corresponding author attests that all listed authors meet the authorship criteria. All authors read and approved the final manuscript.

## FUNDING INFORMATION

This study was supported by the Army Medical University Clinical Medical Research Talent Training Program (2018XLC1005) and the Chongqing Natural Science Foundation (cstc2020jcyj‐msxmX0926).

## CONFLICT OF INTEREST

The authors declare that they have no conflict of interest.

## DATA AVAILABILITY STATEMENT

The data that support the findings of this study are available from the corresponding author upon reasonable request.

## Supporting information


Data S1
