# Supplementary material for: Efficacy and safety of 3‐n‐butylphthalide combined with endovascular treatment in acute ischemic stroke due to large vessel occlusion
Source: CNS Neurosci Ther. 2022 Oct 2;28(12):2298–307. doi: 10.1111/cns.13978 (PMC9627349; doi:10.1111/cns.13978)
Supplement: Supplementary file 1 — Data S1 [file CNS-28-2298-s001.docx]

**3‑n‑Butylphthalide combined with endovascular treatment in acute ischemic stroke due to large vessel occlusion**

**SUPPLEMENTARY APPENDIX**

This appendix has been provided by the authors to give readers additional information about their work.

# 1.Supplemental methods

## Propensity Matching Score Analysis

We performed a 1:1 propensity score matching based on the nearest-neighbor matching algorithm with a caliper width of 0.2 with the usage of SPSS 23.0 (IBM SPSS Statistics).

/VARS

ID = order

TREAT = group

COVS = Hypertension SBP baseline NIHSSonset OTT evt1 evt2 ASTINdich3

ADDLCOVS = OTR

EXACT = Occlusiondich2

/MATCHIT

MATCH=NEAREST

EST =LOGIT

DISCARD = NONE

MORDER = LARGEST

RATIO = 1

CALIPER = .2

/PLOT HISTPLOT JITTERPLOT HISTBAL DOTPLOT INDBAL RESOLUTION = 96

/OUTPUT ALL WIDE.

| **Sample Sizes** | | |
| --- | --- | --- |
|  | Control | Treated |
| All | 1053 | 617 |
| Matched | 599 | 599 |
| Unmatched | 454 | 18 |
| Discarded | 0 | 0 |

| **Overall balance test (Hansen & Bowers, 2010)** | | | |
| --- | --- | --- | --- |
|  | chisquare | df | p.value |
| Overall | 9.438 | 9.000 | .398 |

| **Relative multivariate imbalance L1 (Iacus, King, & Porro, 2010)** | | |
| --- | --- | --- |
|  | Before matching | After matching |
| Multivariate imbalance measure L1 | .971 | .942 |

| **Summary of unbalanced covariates (\|d\| > .25)** |
| --- |
| No covariate exhibits a large imbalance (\|d\| > .25). |

**RGraph**


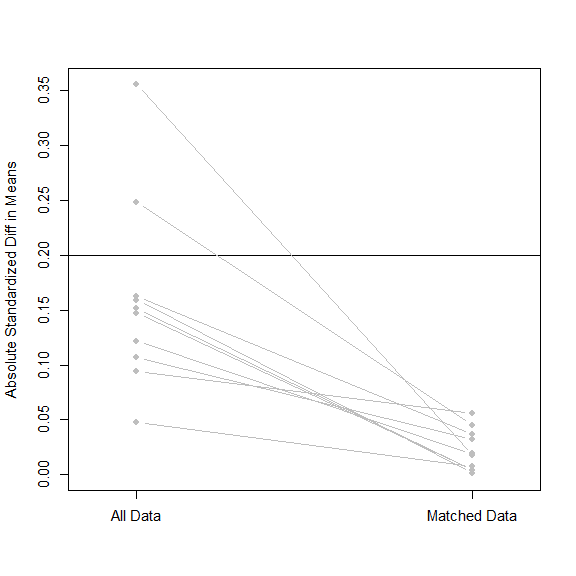


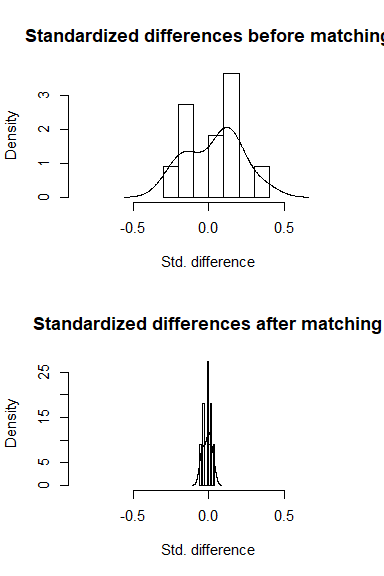


# 2.Supplemental tables

**2.1. eTable 1.** Baseline Characteristics of the Cohort by Occlusion Site

|  | Anterior circulation | | |  | Posterior circulation | | |  |
| --- | --- | --- | --- | --- | --- | --- | --- | --- |
| **Characteristics** | All | NBP | non-NBP | P value | All | NBP | non-NBP | P value |
| Age, years, median (IQR) | 67(58-75) | 67(58-75) | 68(58-76) | 0.225 | 64(56-73) | 64(56-72.5) | 64(56-73) | 0.485 |
| Male sex, n (%) | 57.0% | 57.8% | 56.6% | 0.690 | 74.6% | 72.1% | 76.1% | 0.261 |
| Baseline NIHSS score, median (IQR) | 15(11-19) | 14(10.5-18) | 15.5(12-20) | ＜0.001 | 26(16-33) | 24(14-31) | 28(18-34) | ＜0.001 |
| **ASITN/SIR grade** |  |  |  | 0.020 |  |  |  | 0.313 |
| 0-1 | 40.6% | 35.7% | 43.6% |  | 59.2% | 55.4% | 61.4% |  |
| 2 | 32.3% | 33.6% | 31.5% |  | 26.2% | 28.9% | 24.6% |  |
| 3 | 27.1% | 30.7% | 24.9% |  | 14.6% | 15.7% | 14.0% |  |
| **Baseline ASPECT score, median (IQR)** | 8(7-10) | 8(7-10) | 8(7-10) | 0.351 | 8(7-9) | 8(7-9) | 8(7-9) | 0.222 |
| Blood pressure on admission, median (IQR), mmHg | 146(130-162) | 144(127-160) | 148(130-165) | 0.015 | 150(133-166) | 146(130-162) | 150(134.5-168) | 0.032 |
| **Medical history, No. (%)** |  |  |  |  |  |  |  |  |
| Hypertension | 50.9% | 42.2% | 56.1% | ＜0.001 | 68.6% | 68.0% | 69.0% | 0.804 |
| Hyperlipidemial | 15.8% | 18.0% | 14.4% | 0.099 | 33.8% | 36.1% | 32.5% | 0.334 |
| Diabetes mellitus | 19.4% | 17.6% | 20.5% | 0.219 | 23.3% | 23.0% | 23.5% | 0.881 |
| Smoking | 27.1% | 28.8% | 26.1% | 0.326 | 37.1% | 34.8% | 38.4% | 0.360 |
| Ischemic stroke | 12.1% | 11.9% | 12.3% | 0.833 | 21.0% | 19.3% | 22.0% | 0.397 |
| Atrial fibrillation | 46.9% | 44.7% | 48.3% | 0.247 | 21.3% | 21.3% | 21.3% | 0.996 |
| **Treatment profiles** |  |  |  |  |  |  |  |  |
| Onset to groin puncture, median (IQR), min | 299(225-395.25) | 310(233-416) | 290(220-380) | 0.013 | 330(222-496) | 330(218.5-532) | 330(223-482) | 0.360 |
| Onset to revascularization, median (IQR), min | 365(255-510) | 367(251-519.5) | 361(256-506.5) | 0.848 | 443(328-631) | 440(335.5-671.5) | 450(326-616.5) | 0.217 |
| Groin puncture to revascularization, median (IQR | 95(63.75-135) | 90(60-125.5) | 98(66-141.5) | 0.015 | 104(70-151) | 107(70.5-155.5) | 103(70-145) | 0.534 |
| **Stroke causative mechanism** |  |  |  | 0.068 |  |  |  | 0.706 |
| Large artery atherosclerosis | 40.1% | 39.7% | 40.4% |  | 64.1% | 65.2% | 63.5% |  |
| Cardioembolism | 51.7% | 49.8% | 52.9% |  | 27.3% | 27.5% | 27.3% |  |
| Other | 8.1% | 10.5% | 6.7% |  | 8.6% | 7.4% | 9.2% |  |
| General anesthesia, No. (%) | 75.8% | 77.2% | 75.0% | 0.404 | 59.6% | 59.5% | 59.6% | 0.978 |
| IV Thrombolysis | 30.2% | 29.2% | 30.9% | 0.556 | 18.2% | 19.3% | 17.5% | 0.578 |
| **mTICI score** |  |  |  | 0.096 |  |  |  | 0.497 |
| 0-2a | 13.8% | 11.6% | 15.1% |  | 19.5% | 20.9% | 18.7% |  |
| 2b or 3 | 86.2% | 88.4% | 84.9% |  | 80.5% | 79.1% | 81.3% |  |
| **Medications** |  |  |  |  |  |  |  |  |
| Antihypertensive drugs | 49.8% | 41.3% | 55.1% | ＜0.001 | 67.8% | 67.6% | 67.9% | 0.934 |
| Hypoglycemic durgs | 18.6% | 17.0% | 19.6% | 0.279 | 22.8% | 23.0% | 22.7% | 0.934 |
| Lipid-lowering drugs | 15.4% | 17.6% | 14.0% | 0.107 | 32.5% | 34.2% | 31.6% | 0.497 |
| Anticoagulant drug | 46.0% | 43.7% | 47.4% | 0.220 | 20.3% | 20.2% | 20.3% | 0.979 |
| NBP administered | 38.0% |  |  |  | 36.6% |  |  |  |

IQR, interquartile range; NIHSS, National Institutes of Health Stroke Scale; ASPECTS, Alberta Stroke Program Early CT Score; mTICI, modified thrombolysis in cerebral infarction; IV, intravenous.

**2.2. eTable 2.** Efficacy Outcomes and Safety Outcomes of acute Anterior LVO

| **Characteristics** | All(n=1152) | NBP(n=438) | non-NBP(n=714) | χ2/z Value | P value | Adjusted Value (95%CI) | P Value |
| --- | --- | --- | --- | --- | --- | --- | --- |
| **Primary Efficacy Outcomes** |  |  |  |  |  |  |  |
| Modified Rankin Scale score at 90d, median (IQR) | 3(1-5) | 3(1-4) | 3(2-6) | -4.401 | ＜0.001 | 1.463(1.173-1.824) | 0.001 |
| **Secondary Efficacy Outcomes** |  |  |  |  |  |  |  |
| Modified Rankin Scale score at 90d, No. (%) |  |  |  |  |  |  |  |
| 0-3, No. (%) | 56.2% | 64.2% | 51.3% | 18.362 | ＜0.001 | 1.578(1.171-2.127) | 0.003 |
| 0-2, No. (%) | 42.1% | 47.3% | 39.0% | 7.688 | 0.006 | 1.269(0.952-1.692) | 0.105 |
| 0-1, No. (%) | 26.0% | 29.7% | 23.7% | 4.980 | 0.026 | 1.256(0.917-1.718) | 0.153 |
| **safety outcome** |  |  |  |  |  |  |  |
| **intracranial hemorrhage** |  |  |  |  |  |  |  |
| Symptomatic, No. (%) | 16.0% | 16.0% | 16.0% | 0.000 | 0.994 | 1.038(0.720-1.498) | 0.840 |
| Mortality at 90 days, No. (%)* | 22.7% | 15.1% | 27.4% | 23.450 | ＜0.001 | 0.557(0.389-0.798) | ＜0.001 |

LVO, large vessel occlusion; IQR, interquartile range; CI, Confidence Interval; *All cause death

**2.3. eTable 3.** Efficacy Outcomes and Safety Outcomes of acute Posterior LVO

| **Characteristics** | All(n=656) | NBP(n=244) | non-NBPl(n=412) | χ2/z Value | P value | Adjusted Value (95%CI) | P Value |
| --- | --- | --- | --- | --- | --- | --- | --- |
| **Primary Efficacy Outcomes** |  |  |  |  |  |  |  |
| Modified Rankin Scale score at 90d, median (IQR) | 5(2-6) | 4.5(1-6) | 6(3-6) | -4.23 | ＜0.001 | 1.717(1.254-2.351) | 0.001 |
| **Secondary Efficacy Outcomes** |  |  |  |  |  |  |  |
| Modified Rankin Scale score at 90d, No. (%) |  |  |  |  |  |  |  |
| 0-3, No. (%) | 32.4% | 39.8% | 28.2% | 9.420 | 0.002 | 1.667(1.102-2.524) | 0.016 |
| 0-2, No. (%) | 27.3% | 32.8% | 24.2% | 5.780 | 0.016 | 1.378(0.887-2.140) | 0.154 |
| 0-1, No. (%) | 20.6% | 25.8% | 17.5% | 6.494 | 0.011 | 1.577(0.997-2.496) | 0.052 |
| **safety outcome** |  |  |  |  |  |  |  |
| **intracranial hemorrhage** |  |  |  |  |  |  |  |
| Symptomatic, No. (%) | 6.9% | 1.6% | 10.0% | 16.571 | ＜0.001 | 0.127(0.043-0.377) | ＜0.001 |
| Mortality at 90 days, No. (%)* | 45.6% | 33.6% | 52.6% | 22.496 | ＜0.001 | 0.369(0.244-0.558) | ＜0.001 |

LVO, large vessel occlusion; IQR, interquartile range; CI, Confidence Interval; *All cause death

**2.4. eFigure 1** Subgroup Analysis of mRS at 90 days of the NBP group and the non-NBP group stratified for anterior versus posterior circulation.

|  | P for interaction |
| --- | --- |
| mRS score | 0.867 |
| Anterior LVO |  |
| Posterior LVO |  |
| mRS 0-3 | 0.951 |
| Anterior LVO |  |
| Posterior LVO |  |
| mRS 0-2 | 0.689 |
| Anterior LVO |  |
| Posterior LVO |  |
| mRS 0-1 | 0.427 |
| Anterior LVO |  |
| Posterior LVO |  |
| sICH | 0.001 |
| Anterior LVO |  |
| Posterior LVO |  |
| Mortality | 0.890 |
| Anterior LVO |  |
| Posterior LVO |  |

**2.5 eFigure 2**：Figure shows benefit in the NBP group when compared to the non-NBP group across the 90-day mRS distribution in all subjects with age >= 65 years vs. < 65 years. Threshold for age was chosen at the median.

**2.6. eFigure 3:** Figure shows benefit in the NBP group when compared to the non-NBP across the 90-day mRS distribution in all subjects stratified by sex.

**2.7. eFigure 4:** Figure shows benefit in the NBP group when compared to the non-NBP group across the 90-day mRS distribution in all subjects stratified by baseline NIHSS. Threshold for NIHSS was chosen at the median.

**2.8．eFigure 5 ：**Figure shows benefit in the NBP group when compared to the non-NBP group across the 90-day mRS distribution in all subjects with baseline ASPECTS < 8 vs. >= 8. Six subjects had no ASPECTS score. Threshold for ASPECTS was chosen at median.

**2.9 eFigure 6:** Figure shows benefit in the NBP group when compared to the non-NBP group across the 90-day mRS distribution in all subjects stratified by occlusion site.

**2.10 eFigure 7：**Figure shows benefit in the NBP group when compared to the non-NBP group across the 90-day mRS distribution in all subjects stratified by time from stroke onset to recanalization time (OTR). Threshold for time was chosen at the median.

**2.11. eFigure 8:** Figure shows benefit in the NBP group when compared to the non-NBP group across the 90-day mRS distribution in all subjects stratified by mTICI

**2.12. eFigure 9:** Figure shows benefit in the NBP group when compared to the non-NBP group across the 90-day mRS distribution in all subjects stratified by intravenous thrombolysis（IVT）
